# Professional practice‐related training and organizational readiness for change facilitate implementation of projects on the national core value system in care of older people

**DOI:** 10.1002/nop2.185

**Published:** 2018-07-13

**Authors:** Õie Umb Carlsson, Barbro Wadensten

**Affiliations:** ^1^ Department of Public Health and Caring Sciences Uppsala University Uppsala Sweden

**Keywords:** elder care, nurses, nursing, older people, Sweden

## Abstract

**Aim:**

To explore whether professional training contributed to implementation of the national core value system in practice in care of older people and to identify course participants' perceptions about factors that facilitated or obstructed them in implementing their projects. To identify participants' perceptions concerning factors that facilitate or obstruct implementation.

**Design:**

Descriptive and cross‐sectional.

**Methods:**

Data were retrieved from 451 participants who had completed the course “Understanding and providing leadership based on the national core value system for older people” at Uppsala University during spring semester and autumn semester of 2018. Quantitative and qualitative data were obtained using a web‐based questionnaire.

**Results:**

The results showed that the majority (73%) of project plans were initiated, although not always completed and sometimes interrupted. Organizational readiness in terms of management supporting and prioritizing these plans were two principle facilitators chosen by half of the respondents and consequently the absence of these factors was an obstacle. In addition, successful implementation required dedicated co‐workers and certain resources, such as time and funds. Surprisingly, factors related to the project leader were reported to be of limited importance.

## 1 INTRODUCTION

The present article concerns educational efforts aimed at managers in care of older people and their opportunities to implement the project plans they made as part of a course. The aim of the course was to teach managers about a change in Swedish law concerning “the national core value system for care of older people” and to encourage them to work, at their workplaces, with the changes required to implement the new ideas in the legal text. An earlier study (Umb Carlsson, Källberg, & Wadensten, 2015) investigated all of the managers' project aims and found that they sought to develop and improve measures that facilitate older persons' participation in decision‐making. Four different categories of projects were identified: management and organization (*N = *351, 43.9%), professional development (*N = *133, 16.6%), working methods (*N = *289, 36.1%) and user participation (*N = *27, 3.4%). A detailed description of the categories has previously been presented (ibid). Project plans were based on values in line with the national core value system, taking the older person's perspective into account when planning and implementing support and care on various levels: the organizational, activity and individual level.

### 1.1 Background

In Sweden, the Social Services Act, SoL (SFS, 2001:453), regulates residents' rights to economic and social support as well as municipalities' obligations in relation to residents in their jurisdiction. The municipalities make judgments concerning older people's right to home‐care services in ordinary housing and in nursing homes. During recent decades, as in many other countries, there has been a paradigm shift towards more person‐centred care. Thus, in 2011, an add‐on was made to the SoL (SFS, 2001:453). The new text relates to governmental priorities and states that older people have the right to lead a life characterized by dignity and well‐being (Chap. 5. 4§ SoL). This new text is called “the national core value system for elderly care.”

The national core value system is central to care of older people and shows the ethical values and norms that should form the basis for working procedures and activities. The core value system covers seven components: self‐determination, personal safety, meaningfulness and coherence, respect for personal privacy and integrity, person‐centred care and user involvement, good treatment and high quality in care. It clarifies values and can be seen as an ethical approach that should involve the entire organization, so as to ensure tailored services and care for individual needs.

However, many older people experience limited autonomy and self‐determination in everyday life (Hellström & Sarvimäki, 2007; Ottenvall Hammar, Dahlin‐Ivanoff, Wilhelmson, & Eklund, 2015). To familiarize leaders working in care of older people with the new text, the National Board of Health and Welfare was commissioned to start a course for managers, nurses and rehabilitation professionals. The objective was to increase their competence and help them apply the national core value system in the services they provide. The course was entitled: “Understanding and providing leadership based on the national core value system for older people” (7.5 credits).

Based on earlier positive experiences of blended learning among similar groups (Arving, Wadensten, & Johansson, 2013; Atack, 2003; Sung, Kwon, & Ryu, 2008), the same approach was chosen. Web‐based education, including various kinds of e‐learning, was combined with conventional face‐to‐face communication. A detailed description of the training programme has been previously reported (Umb Carlsson et al., 2015). The course was offered on a 25% of full‐time basis. The intention was for the participants to continue working at their regular workplaces during the course. Previous studies have suggested that training of practitioners ought to be related to use in practice and take into account the context of implementation (Manuel, Mullen, Fang, Bellamy, & Bledsoe, 2009). The combination of work and training is intended to aid in implementing the new knowledge in everyday practice.

The course had four main areas: the national core value system, ethics and humanism, communication and knowledge of improvement work. The aim was to connect theory and practice, provide an arena for reflection and teach participants how the core value system can be concretized and used to achieve systematic improvements on an operational and organizational level. Improvement work was based on the Y‐model, which describes a process alternating between current mode and intended mode, change needs and change options and an action plan until the change to be implemented is chosen (Lundeberg, 1993). Thus, improvement work was not considered linear, but as shifting back and forth during the change process (cf. Källberg, 2013). It has been suggested that applying service improvement projects in education and training has a positive effect on learning (Baillie, Bromley, Walker, Jones, & Mhlanga, 2014). Thus, the course ended with an assignment to create a project plan; participants were to describe an improvement they wished to implement at their own workplace. The improvement was to be related to the national core value system. In the project plan, the participants motivated their proposal for improvement and reflected on how it related to the national core value system. In addition, participants described how the plan would be implemented, who would be involved and how improvement efforts would be evaluated. The kinds of projects designed fell into four different categories: management and organization, professional development, working methods and user participation (Umb Carlsson et al., 2015). About 900 persons started the course during the first two semesters it was offered. Of those, 780 persons completed the course successfully.

Implementing change and improving the quality of care involve a difficult, complex and demanding process (Rycroft‐Malone, 2004); they do not follow prescribed and linear paths (Rycroft‐Malone et al., 2002). Many studies have pointed out various factors that obstruct implementation (McCormack et al., 2002; Retsas, 2000; Røsstad et al., 2015). The complexities of service settings, including competence of staff members, quality of administrative leadership, organizational norms and expectations, may hamper work towards change (Glisson, 2002). In a literature review, Thompson, Estabrooks, and Degner (2006) pointed out the importance of interpersonal relations between professionals when implementing innovations in the health sector. Thus, successful quality improvement requires organizational readiness (Weiner, 2009).

Continuing training is of great importance and a principal method for professional development in care of older people. The challenge of implementing the skills and knowledge provided through professional training in practice is well acknowledged in the social services field. Nevertheless, to the best of our knowledge, there is no previous research on how educational efforts aimed at care of older people managers affect implementation of government policy in practice.

### 1.2 Rationale for the study

Typically, one objective of professional training is that the knowledge gained be implemented and, thus, affect everyday practice. The course on the national core value system encouraged participants to identify areas in need of improvement at their workplaces in relation to the basic value system as well as to write an action plan for implementation of such improvements. In this light, it is of value to study to what extent participants' ideas about possible improvements were implemented and identify facilitators of and obstacles to implementation. Findings from such a study could support implementation of government policy in everyday practice.

### 1.3 Overall aim and specific study questions

The overall aim of the present study was to explore whether professional training contributed to implementation of the national core value system in practice in care of older people and to identify course participants' perceptions about factors that facilitated or obstructed them in implementing their projects. Specific study questions were:

- To what extent were the projects implemented?
- What factors facilitated implementation of their projects?
- What factors obstructed implementation of their projects?
- To what extent were the projects evaluated?

## 2 METHOD

### 2.1 Design

The survey‐based study was descriptive and cross‐sectional (Polit & Beck, 2011). Quantitative and qualitative data were obtained using a web‐based questionnaire. Participants who filled out the questionnaire had previously completed a professional training course.

### 2.2 Sample

All participants who had completed the course “Understanding and providing leadership based on the national core value system for older people” at Uppsala University during spring semester and autumn semester of 2012 were invited to participate in the study (*N* = 780). The invited participants worked in the community‐based care of older people system at various locations in Sweden and attended the course as a part of their employment. They had different professions (unit managers, care managers, nurses responsible for medical issues and rehabilitation professionals) and thus, different educational backgrounds. All had responsibilities regarding implementation of the national core value system in practice.

### 2.3 Instrument

A study‐specific web‐based questionnaire was developed by the authors to gather information on implementation of the projects course participants had planned within the framework of the course. Face validity regarding the questionnaire's relevance and wording was discussed with researchers and teachers involved in the course. Minor adjustments were made to clarify the wording and response alternatives. The questionnaire included five questions asking about: (a) whether the project had been commenced and completed; (b) whether commenced or completed projects had been evaluated; (c) whether the reason for not implementing the project had been discussed and what factors had, according to the respondent; (d) facilitated; and (e) obstructed implementation of project plans. The response alternatives for the first question were: yes, commenced and will be completed, commenced but interrupted and no. The response alternatives for the second and third questions were: “yes”, “no but planned”, “no”. It was possible to give a free‐text answer and specify how the evaluation/discussion was conducted. The fourth and fifth questions asked respondents to choose three important facilitating and obstructing factors from a list of 12 alternatives. In addition, it was possible to specify a factor that was not on the list, i.e. to provide a free‐text answer.

### 2.4 Data collection

The data were collected in 2013, i.e. one year after completion of the course, using SurveyXact, an online tool. A link to the questionnaire was sent to potential respondents via e‐mail; two reminders were also sent out. The respondents´ e‐mail addresses were obtained from the course participant lists from 2012. A cover letter with information about the study, voluntary consent and anonymity was included in the e‐mail. Returning a completed questionnaire was considered written consent.

Information on demographic data regarding age, gender, training period and occupational category was obtained from the course participant lists from 2012. The web‐based tool enabled identification of respondents, but not the answers they gave.

### 2.5 Analysis

Quantitative data were entered into a document and transferred to an SPSS file. The statistical analysis was processed to generate descriptive statistics using IBM SPSS statistics version 22. Analyses of frequency distributions, means, standard deviations and ranges were used to characterize the demographic data of respondents and non‐responders.

Free‐text answers regarding facilitating and obstructing factors for implementation were analysed separately through directed content analysis (Hsieh & Shannon, 2005). The texts were read and reread several times and meaning units were identified. Most free‐text answers were treated as separate meaning units, although a few were divided into separate meaning units. Then, meaning units were grouped into categories using the questionnaire list of facilitating and obstructing factors (questionnaire items 4 and 5) as initial coding categories. Texts that could not be coded into one of these categories were identified to determine whether they represented a new category or a nuance of an existing one. Analyses were performed by the first author and discussed with the second author until consensus was reached.

#### 2.5.1 Ethical considerations

In accordance with Swedish laws and regulations, an ethics committee was not required (SFS, 2003:460). However, the recommendations for research ethics in Sweden were followed, as all respondents received written and oral information about the study, stating that participation was voluntary and that their responses would be treated confidentially. Moreover, written informed consent was collected from respondents (Codex, 2016). To achieve anonymity, responses cannot be linked to an individual respondent.

## 3 RESULTS

Just over half (58%) of the invited course participants completed and returned the web‐based questionnaire. Their mean age was 49.5 years (*SD*. 8.5, range 24–68 years). The mean age of non‐respondents was 47.3 years (*SD* 9.0, range 26–66 years). An overwhelming majority were women (98% of 451), which reflects the gender distribution among course participants. No significant differences related to training period were identified. Thus, results are displayed as a whole and not divided into spring semester and autumn semester, respectively. Most answers were provided by unit managers (65% of 451). The average response rate was 50% across all professional categories: 52% of unit managers, 50% of social workers, 66% of nurses, 61% of developers and 68% of others (including paramedical staff, ombudsmen, teachers and consultants). There were no missing data due to non‐responses on single questions. Because data collection was anonymous, it was not possible to connect individual answers with occupational categories and project plans.

### 3.1 Extent of project implementation

Analyses of the 451 completed questionnaires indicate that the majority (73%) of project plans were commenced, although not always completed and in some cases interrupted (Table 1).

**Table 1:** Extent of project implementation

Extent of implementation	*N* (%)
Completed	96 (21)
Commenced, will be completed	189 (42)
Commenced, interrupted	43 (10)
Neither completed nor commenced	103 (23)
Do not know	20 (4)
Total	451 (100)

### 3.2 Facilitating factors

The answers to the question on which factors had facilitated implementation of project plans were diverse and all alternatives on the list were chosen by one or more respondents. Management support and dedicated co‐workers were principle facilitators, chosen by half of the respondents (Table 2). Several different combinations of facilitators were reported; however, analyses did not reveal any combination to be of particular importance. This is illustrated by the fact that the most frequently reported combination, management support and dedicated co‐workers, was only reported by 25% (*N* = 285). Analyses of free‐text answers did not add further facilitators but specified the alternatives on the list. It may be noted that factors related to the project leader—such as knowledge, dedicated time and tutoring—were of less significance.

**Table 2:** Facilitators of implementation of project plans

Facilitators	Completed or commenced projects
	*N* (%)
Dedicated co‐workers	146 (51)
Management support	143 (50)
Decided and planned previously	98 (35)
Presence of an enthusiast	69 (24)
Resources were available for implementation costs	60 (21)
Political directives	58 (20)
Time was set aside for co‐workers	55 (19)
The project leader had sufficient knowledge	48 (17)
Time was set aside for a project leader	42 (13)
The project leader was provided further education	29 (9)
A tutor or mentor supported the process	24 (7)
Other	36 (11)
Total (*N* = 285)	

### 3.3 Obstructing factors

Respondents who reported that their project either was not commenced or was interrupted specified three main reasons for obstructed implementation (Table 3). Obviously, implementation was impeded or prevented if the project had received no support from management. However, management having a positive view of the project was not considered sufficient; the project had to be given priority over other projects. A further obstacle was if insufficient resources, such as time and funds, had been allocated to the project. Reorganization and personnel turnover were other factors that, according to the respondents, obstructed implementation, although not to the same extent as the factors mentioned above. Just as for the facilitating factors, obstructing factors related to the project leader were reported to be of limited importance.

**Table 3:** Obstructions to implementation of project plans

Obstructing factors	Interrupted or not commenced projects
	*N* (%)
The management was positive but prioritized other projects	55 (38)
Lack of time on the part of the project leader	47 (32)
No resources were available for implementation costs (material, facilities etc.)	43 (29)
I changed employment and no one else drove the project	43 (29)
Co‐workers who were interested had a too much else to do	42 (29)
The project was never meant to be implemented	40 (27)
No support from management	35 (24)
There was no project leader with sufficient knowledge	12 (8)
Reorganization	11 (8)
The project leader was too isolated and had no tutor or mentor	10 (7)
No co‐workers were interested	9 (6)
Co‐workers opposed the project	4 (3)
The politicians said no	4 (3)
Other	6 (4)
Total (*N* = 146)	

### 3.4 Evaluations

Only 15% of the projects that had been completed or commenced (*N* = 285) had been evaluated or systematically followed up. However, evaluation or follow‐up was planned for another half, i.e. respondents expected that evaluation would be performed for most implemented projects (69%).

According to the respondents, data for evaluations and follow‐ups were obtained using a variety of recognized measures (face‐to‐face interviews, group interviews, questionnaires and reviews of existing individual user plans) as well as based on information provided by older people, relatives and personnel. For the most part, evaluations and follow‐ups were conducted by the concerned authority and universities.

For interrupted or not commenced project plans (*N* = 146), the figures were reversed and no evaluations or follow‐ups had been conducted or planned for 79%. Thus, besides the respondents´ experiences, there is no knowledge as to why the project plans were not implemented.

## 4 DISCUSSION

The present study was based on questionnaire data from 451 respondents who had prepared a project plan within the framework of a blended course on the national core value system for older people in Sweden. Most answers were provided by unit managers (65% of 451), which was expected because this was the largest occupational category. According to the respondents, most of the project plans (73%) had been commenced in everyday practice, although not always completed and in some cases interrupted. Organizational readiness in terms of management support, in conjunction with dedicated co‐workers, was identified as a principle facilitating factor. However, even if management favoured the project, it had to be given priority over other projects. In addition, lack of allocated resources was a frequent obstructing factor for implementation of project plans.

The present study indicated that co‐workers, in addition to management, played a crucial role. Engagement and allotted time were facilitating factors related to co‐workers, while the opposite —disinterest, counteracting and lack of time—were listed as obstructing factors. To our knowledge, there is limited documentation of the role played by co‐workers in implementing new knowledge in everyday practice following professional training in social care of older people. However, in the field of implementation of interventions, numerous studies have argued that successful implementation of an intervention requires that several employees be included and that they experience the change as useful and valuable (Murray, Douglas, Girdley, & Jarzemsky, 2010; Røsstad et al., 2015). Lack of coherence among employees may also be an important challenge to implementation (Bamford, Heaven, May, & Moynihan, 2012). It is reasonable to believe that these findings are also applicable to implementation of the output of professional training in everyday practice. Nevertheless, more studies on this topic are necessary.

Many studies have investigated the problems associated with implementing knowledge in practice and found insufficient time for implementing new ideas to be a crucial factor (Bryar et al., 2003; Carlson & Plonczynski, 2008; Gerrish & Clayton, 2004; Retsas, 2000). Our study shows that only a small proportion of respondents considered allocated time for the project leader and co‐workers to be important facilitating factors. In contrast, lack of time was reported to be a central reason for projects not being implemented.

One purpose of professional training is for theoretical knowledge to contribute to quality development in everyday practice (Schatzki, 2002; Umb Carlsson et al., 2015). The present study showed that a quarter of the project plans were never commenced. For several projects, no resources were allocated to implementation and in some cases implementing the project was never the intention. This raises questions about why employees are offered training and points to the need for mutual interaction between professional training and a facilitative environment. Workplaces do not exist in isolation but are part of and dependent on the environmental context; they may be described as “practice‐arrangement bundles” (Schatzki, 2010) or as an organized nexus of activities (Groves & Rönnerman, 2012). Organizational obstacles, such as lack of interest on the part of management, have been reported in several studies (Bryar et al., 2003; Hannes et al.., 2007; McCormack et al., 2002; Rycroft‐Malone et al., 2002). It has been argued that one prerequisite for organizational support is that interventions match the priorities and strategies selected by management (Duner, Blomberg, & Hasson, 2011; Røsstad et al., 2015) and that this match (or lack thereof) may serve as a way to predict and control the work environment (Habermas, 1971). In our study, this was demonstrated by the finding indicating that management support was a principle feature, but for successful implementation to occur, the project had to be given priority over other projects. Unit manager was the largest professional category to complete the questionnaire. It is therefore surprising that lack of interest on the part of other managers and lack of resources and priority over other projects were the main obstacles reported. Such obstacles should not be relevant for managers, because these are factors they can control by setting aside resources and making priorities themselves. However, organizational readiness for change is presumably of more importance. Here, Weiner's definition of organizational readiness for change, ORC (Weiner, 2009), may help us understand this finding, in that it indicates that projects were not firmly rooted in the organization. Further, Weiner suggested that successful implementation requires that professionals at all hierarchical levels share perceptions of readiness for the specific change effort. Another prerequisite is that organization members value the change and find it important (collective commitment), as well as that they experience the existence of sufficient resources and cognitive capability to implement the changes (collective efficacy). The present findings make visible the practical challenges of developing and implementing projects that encompass a shift in perspectives, attitudes and behaviour. The results point out that it is not sufficient that professionals is offered relevant tools for applying the national core value system in their workplaces, but there is also a need for mutual interaction between the professional training and a facilitative environment and organization.

When trying to understand the impact of facilitators of and obstacles to implementation of the national core value system in the form of a project, applying implementation theory is useful. One of the classic theories is Rogers' (2003) diffusion of innovation theory. According to Rogers, information exchange concerning an innovation is at the heart of the diffusion process. Moreover, Rogers claimed that the probability of adopting an innovation increases with increased observability (visibility), relative advantage associated with using the innovation, lack of complexity (understandability), compatibility with extant values and trialability (potential to be acquired “piece by piece”). These are not the only qualities of innovations that affect adoption rates, but they are important, perhaps the most important, characteristics explaining them. Previous results have shown that the training stimulated thoughts and ideas related to the national core value system (cf. Umb Carlsson et al., 2015) and that most participants wanted to implement their ideas. The present results revealed that environmental factors could act as facilitating or obstructing factors. Introducing the national core value system implied a paradigm shift challenging prevailing values and working methods, from regarding older people like passive recipients of social care to treating them as active subjects, considering their preferences and wishes. Realizing this shift in attitudes and behaviour is complex and much more complicated than introducing a new bandage or catheter. Above all, it takes time. The national effort is unique and provides a base but has to be complemented with discussions and reflections in everyday practice. In addition, in the blended education course, it would have been valuable to dedicate more time and space to the need to anchor the project in the organization (Weiner, 2009) and, e.g. to connect this to Rogers' diffusion of innovation theory (Rogers, 2003). Further, it would have been valuable if the course had included a day of follow‐up with reflection on experiences of implementation efforts and had connected this to implementation theory and the use of checklists, such as those developed by Flottorp et al. (2013). The PARIHS framework (Kitson et al., 2008; Kitson, Harvey, & McCormack, 1998; Rycroft‐Malone et al., 2002) could also have been used as a guide when implementing new ideas. In addition, it might have been valuable to dedicate more time and space to the need for evaluation, regardless of whether or not a project was implemented.

### 4.1 Strengths and limitations

The survey was an online survey with a response rate of 58%, which is lower than the expected rate. The attrition rate is based on the number of course participants in 2012. The questionnaire was sent to e‐mail addresses obtained from the course participant lists from 2012, but the number of respondents who received the survey is not known. Factors such as changed employment, absence due to sick leave, vacation or parental leave and spam filters may have resulted in the e‐mail message not reaching all recipients. However, the response rate can be considered acceptable for web‐surveys (Nulty, 2008; Richardson, 2005).

Data collection was anonymous, which is a strength, but may also be a disadvantage. It is not possible to connect individual answers with occupational categories and review if the multidisciplinary nature of respondents has affected the results. The results are based on respondents' self‐reported perceptions, collected using a face validated questionnaire developed for the present study. This may limit the reliability of our findings, owing to the risk that the respondents gave a more positive picture of their implementation than would have been revealed by other data collection methods. A review of projects that have been implemented in the municipalities might have provided another perspective. Nonetheless, the present study included the majority of course participants and the results are in accordance with findings from previous research.

## 5 CONCLUSION

The present findings indicate that increased knowledge and awareness of the national core value system among professionals are not sufficient to support implementation of the government policy in practice. The findings point to the need for organizational readiness if change is to occur. One purpose of professional training in care of older people is for theoretical knowledge to contribute to quality development in practice. The study indicates that although the specially focused training may have contributed to increased knowledge among professionals, this knowledge gain was not sufficient to promote change in practice. The findings point to the need for mutual interaction between professional training and a facilitative environment. Management supporting and prioritizing project plans are principle facilitators and are more important than factors related to the project leader.

## CONFLICT OF INTEREST

None.
